# Preservice Mathematics Teachers’ Problem-Solving Self-Efficacy, Critical Thinking Disposition, and Mathematical Creativity Self-Efficacy: A Cross-Sectional Path Analytic Study

**DOI:** 10.3390/jintelligence14100271

**Published:** 2026-10-21

**Authors:** Murat Genç, Mustafa Akıncı, İlhan Karataş, Özgür Murat Çolakoğlu

**Affiliations:** 1 Department of Mathematics and Science Education, Zonguldak Bülent Ecevit University, Zonguldak 67300, Turkey; mustafa.akinci@beun.edu.tr (M.A.); ilhankaratas@beun.edu.tr (İ.K.); 2 Department of Educational Sciences, Zonguldak Bülent Ecevit University, Zonguldak 67300, Turkey; omuratcolakoglu@beun.edu.tr

**Keywords:** problem-solving self-efficacy, critical thinking disposition, mathematical creativity self-efficacy, observed-variable path analysis

## Abstract

Developing flexible thinking, generating alternative solution strategies, and producing original ideas are increasingly emphasized in mathematics education. This study examines the relationships among preservice elementary mathematics teachers’ problem-solving self-efficacy, critical thinking disposition, and mathematical creativity self-efficacy. Drawing on a theoretically informed framework, it specifically investigates whether critical thinking disposition statistically mediates the association between problem-solving self-efficacy and mathematical creativity self-efficacy. Data were collected from 554 preservice elementary mathematics teachers. Analyses included descriptive statistics, Pearson correlations, and bootstrap-based observed-variable path analysis to obtain robust estimates of direct and indirect associations. Findings indicated that positive problem-solving self-efficacy was strongly associated with critical thinking disposition, which was, in turn, positively associated with mathematical creativity self-efficacy. Critical thinking disposition also statistically mediated the association between problem-solving self-efficacy and mathematical creativity self-efficacy. A reverse-direction model showed comparably good overall fit, indicating that the observed data were also compatible with an alternative directional ordering. These findings suggest that problem-solving self-efficacy and critical thinking disposition represent complementary cognitive-affective constructs that are empirically associated with perceptions of mathematical creativity. Given the cross-sectional design, the findings should not be interpreted as evidence of causal direction. The results instead provide empirical support for a theoretically motivated model of the associations among problem-solving self-efficacy, critical thinking disposition, and mathematical creativity self-efficacy.

## 1. Introduction

The central paradigm of contemporary mathematics education extends beyond equipping students with procedural and algorithmic competencies. It aims to cultivate individuals who can generate strategies in complex situations, think flexibly, and justify their arguments within a coherent logical framework (National Council of Teachers of Mathematics [NCTM], 2020a). At the core of this objective lies problem solving, which is not merely a cognitive activity but also an affective process shaped by individuals’ self-efficacy beliefs regarding their capabilities when confronting challenges. As implicitly emphasized in George Polya’s theory of problem solving (Polya, 1957), each stage, ranging from problem comprehension to plan implementation and retrospective evaluation, is closely associated with persistence and beliefs about one’s capabilities (Bandura, 1997; Schoenfeld, 2016). Accordingly, in the development of advanced mathematical thinking, problem-solving self-efficacy can be understood as a motivational resource associated with individuals’ willingness to engage in cognitively demanding problem-solving processes.

The search for alternative approaches and the capacity to move beyond routine procedures constitute the foundation of mathematical creativity (Leikin, 2009). For students or preservice teachers to demonstrate mathematical creativity in terms of fluency, flexibility, and originality, they require a disposition toward critical thinking that enables them to evaluate possibilities, systematically question inferences, and select functional solutions (P. Facione, 2015). A review of the literature indicates that problem solving, creativity, and critical thinking are frequently conceptualized as interrelated competencies that collectively support mathematical proficiency. However, empirical evidence explaining how problem-solving self-efficacy is associated with mathematical creativity self-efficacy through critical thinking remains limited. In particular, there is a need for analytical models that examine how these interrelated cognitive-affective variables are connected within an integrated framework (Z. Liu et al., 2025; Tasgin & Dilek, 2023).

Despite extensive research examining self-efficacy, critical thinking, problem solving, and creativity, much of the existing literature has investigated these constructs through isolated or pairwise relationships. Consequently, less attention has been devoted to understanding how broader efficacy beliefs may be associated with domain-specific creative efficacy through an intermediate cognitive disposition. The present study addresses this conceptual issue by proposing and testing a theoretically grounded path model in which problem-solving self-efficacy is associated with mathematical creativity self-efficacy through critical thinking disposition. Rather than introducing new constructs, the study seeks to clarify how previously established constructs may operate together within a coherent cognitive-affective framework and whether the proposed indirect association is empirically supported.

Importantly, the proposed sequence is derived from theoretical considerations rather than from statistical optimization. Drawing on Social Cognitive Theory (Bandura, 1997), efficacy beliefs are viewed as motivational resources that influence individuals’ willingness to engage in cognitively demanding activities. Consistent with Dual-Process Theory (Evans & Stanovich, 2013), individuals with stronger confidence in their problem-solving capabilities may be more willing to engage in effortful, reflective, and analytic thinking, which is conceptually related to a stronger critical thinking disposition. In turn, creativity research suggests that generating mathematically original solutions requires not only idea generation but also systematic evaluation and refinement of those ideas (Amabile, 1996; Runco & Jaeger, 2012). From this perspective, critical thinking disposition may provide a theoretically plausible cognitive mechanism through which broader efficacy beliefs are associated with domain-specific mathematical creativity self-efficacy. This rationale does not imply that critical thinking disposition is necessarily temporally caused by problem-solving self-efficacy; rather, it provides a theoretical basis for examining their hypothesized structural association within the present cross-sectional design.

The proposed sequence also requires consideration of the conceptual distinction between relatively broad efficacy beliefs and domain-specific creative efficacy. Problem-solving self-efficacy concerns individuals’ perceived capability to manage and solve challenging problems, whereas mathematical creativity self-efficacy concerns perceived capability to generate and work with mathematically creative ideas. Critical thinking disposition may constitute a theoretically meaningful link between these forms of self-belief because it reflects an orientation toward questioning assumptions, examining alternatives, evaluating evidence, and engaging in reflective judgment (P. Facione, 2015). In this sense, the proposed framework is intended not simply to rearrange familiar variables but to examine whether a critical cognitive disposition helps explain the statistical association between a broader problem-solving efficacy belief and a more domain-specific creative efficacy belief. At the same time, reciprocal and alternative orderings remain theoretically plausible. For example, mathematical creativity self-efficacy may be associated with greater critical engagement, or critical thinking disposition may be associated with both forms of self-efficacy. Therefore, the proposed sequence is treated as a theoretically motivated specification to be examined against plausible alternative directional models.

Preservice teachers’ perceptions of problem-solving self-efficacy, critical thinking disposition, and mathematical creativity self-efficacy are particularly relevant to mathematics teacher education because these beliefs and dispositions may be reflected in how prospective teachers approach mathematical tasks and conceptualize opportunities for creative mathematical engagement. Low problem-solving self-efficacy or weakly developed critical thinking dispositions may be associated with reduced willingness to engage with complex and open-ended mathematical situations. Although prior studies have examined relationships among these variables, the statistical role of critical thinking disposition in the association between problem-solving self-efficacy and mathematical creativity self-efficacy remains unclear. Addressing this gap, the present study investigates the relationships among preservice elementary mathematics teachers’ problem-solving self-efficacy, critical thinking disposition, and mathematical creativity self-efficacy through observed-variable path analysis, examining both the direct association of problem-solving self-efficacy with mathematical creativity self-efficacy and its indirect statistical association through critical thinking disposition. The study therefore focuses on whether the proposed theory-informed structural representation is empirically consistent with the observed covariance structure and whether it provides greater empirical consistency than theoretically plausible reverse-direction specifications.

Accordingly, the study sought to address the following research questions:

- What are the levels of preservice elementary mathematics teachers’ problem-solving self-efficacy, critical thinking disposition, and mathematical creativity self-efficacy?
- Are the relationships among problem-solving self-efficacy, critical thinking disposition, and mathematical creativity self-efficacy statistically significant?
- What are the direct and indirect associations between problem-solving self-efficacy and mathematical creativity self-efficacy, and is critical thinking disposition statistically associated with the indirect pathway between these constructs?
- To what extent is the proposed path model empirically consistent with the observed relationships among problem-solving self-efficacy, critical thinking disposition, and mathematical creativity self-efficacy relative to plausible alternative model specifications?

## 2. Related Literature and Conceptual Framework

### 2.1. Problem-Solving Self-Efficacy

Problem solving constitutes one of the fundamental components of mathematics curricula from preschool through secondary education. Contemporary curricula aim to foster students’ abilities to generate new mathematical situations, solve problems across diverse contexts, develop and adapt strategies, and monitor and evaluate the problem-solving process (National Council of Teachers of Mathematics [NCTM], 2020a, 2020b). However, success in problem solving depends not only on cognitive competencies but also on individuals’ beliefs about their capabilities to engage successfully in problem-solving activities (Hoffman & Schraw, 2009).

According to Bandura (1997), self-efficacy influences academic functioning by shaping individuals’ beliefs about their capacity to organize and execute the actions required to attain desired outcomes. In problem-solving contexts, such beliefs are particularly relevant because challenging mathematical tasks often require sustained effort, strategic flexibility, and persistence in the face of difficulty. A growing body of research indicates that self-efficacy is associated with strategy selection, persistence, and motivation during problem-solving activities. Path analysis studies have revealed positive relationships between self-efficacy and both mathematics achievement and problem-solving performance (Pajares & Kranzler, 1995; Pajares & Miller, 1994). Furthermore, individuals with higher levels of self-efficacy tend to employ more effective strategies and demonstrate greater effort and perseverance when confronted with challenging problems (Hoffman & Schraw, 2009). Wang et al. (2024) reported that students’ self-efficacy was positively associated with problem-solving disposition, both directly and indirectly through metacognitive and critical thinking dispositions. Collectively, these findings indicate that problem-solving self-efficacy is an important motivational correlate of individuals’ engagement with problem-solving processes.

### 2.2. Mathematical Creativity

Creativity is generally defined as the production of ideas that are novel, original, and useful (Runco & Jaeger, 2012), whereas mathematical creativity refers specifically to the generation of original and functional solutions to mathematical problems (Liljedahl & Sriraman, 2006; Sriraman, 2005). This concept is closely associated with the ability to produce multiple solutions and develop diverse strategies during both problem-solving and problem-posing processes (Leikin, 2009; Silver, 1997). Mathematical creativity contributes to the development of students’ deep understanding, problem-solving competence, and mathematical reasoning skills (Leikin & Sriraman, 2022), and it is widely regarded as an important educational goal for fostering the mathematical potential of all learners (Sipahi & Bahar, 2025).

The conceptualization of mathematical creativity varies across theoretical and measurement frameworks. Kozlowski et al. (2019), for example, conceptualized mathematical creativity through four dimensions: fluency, flexibility, originality, and elaboration. However, fluency, flexibility, and originality are particularly prominent dimensions in the literature on mathematical creativity (Beghetto, 2017; Imai, 2000; Silver, 1997; Sriraman & Haavold, 2017). Fluency refers to the quantity of ideas generated, flexibility denotes the use of varied solution strategies, and originality represents the production of novel or uncommon approaches (Shoimah et al., 2018). Elaboration, by contrast, refers to the development, refinement, or extension of existing ideas. Although elaboration represents a potentially relevant aspect of creativity, the instrument employed in the present study operationalizes mathematical creativity self-efficacy through fluency, flexibility, and originality. This three-dimensional operationalization is consistent with the dimensions most frequently emphasized in systematic reviews of mathematical creativity (Sipahi & Bahar, 2025). Accordingly, elaboration was not treated as a separate dimension in the present study because it was not included in the measurement instrument used to assess participants’ mathematical creativity self-efficacy. The present study therefore does not claim to capture the full range of possible dimensions of mathematical creativity; rather, it focuses specifically on preservice teachers’ perceived efficacy in generating multiple ideas, employing varied strategies, and producing original mathematical approaches. Nijstad et al. (2010) suggest that creativity emerges through two complementary cognitive mechanisms, cognitive flexibility and cognitive persistence, through which dispositional and situational factors shape the generation of original and appropriate ideas across these dimensions.

### 2.3. Critical Thinking

Critical thinking refers to the strategic, deliberate, and iterative use of reasoning skills in individuals’ decision-making and problem-solving processes (P. A. Facione, 1990; Jablonka, 2020). Halpern (2013) conceptualizes this construct as a set of goal-directed and logical cognitive skills that increase the likelihood of achieving desired outcomes. This process involves complex operations such as drawing inferences, estimating probabilities, and critically evaluating judgments. For effective critical problem-solving performance, individuals need relevant domain knowledge, the capacity to evaluate inductive and deductive propositions, and a cognitive disposition toward engaging in such processes (N. C. Facione & Facione, 1996). Contemporary literature confirms that critical thinking enhances the quality of decision-making across a wide range of domains, from health to finance, and is considered one of the core outcomes of modern education (Altun & Yildirim, 2023; Butler, 2024).

Within mathematics education, critical thinking plays a crucial role in integrating mathematical reasoning with real-life applications (Jablonka, 2020). Empirical research indicates that critical thinking functions as an important variable supporting academic competence (Tasgin & Dilek, 2023) and that interventions implemented in problem-based learning environments can substantially enhance these skills (Y. Liu & Pásztor, 2022). As a comprehensive construct, critical thinking encompasses interconnected components including interpretation, analysis, inference, evaluation, explanation, and self-regulation (P. Facione, 2015). Beyond these cognitive skills, the disposition to engage in such processes is particularly relevant when individuals must examine alternatives, question assumptions, and evaluate the adequacy of potential solutions.

### 2.4. Relationships Among Problem-Solving Self-Efficacy, Critical Thinking, and Mathematical Creativity Self-Efficacy

Students’ affective perceptions and self-efficacy beliefs regarding mathematical problem solving play an important role in shaping their cognitive engagement with and approaches to non-routine tasks. It is essential to distinguish between mathematical creativity as a performance-based construct, referring to demonstrated creative performance or output, and mathematical creativity self-efficacy (MCSE) as a perceptual construct, referring to individuals’ confidence in their capacity to think and act creatively in mathematics. Whereas demonstrated mathematical creativity reflects actual cognitive performance (Leikin & Lev, 2013), mathematical creativity self-efficacy represents individuals’ perceptions of their capability to engage in creative mathematical activities, including initiating, persisting in, and exploring alternative strategies during mathematical problem-solving processes (Z. Liu et al., 2025). Although creative ability and creative self-efficacy may interact as complementary components within the broader domain of mathematical creativity (Bicer et al., 2020; Sadak et al., 2022), the present study focuses specifically on mathematical creativity self-efficacy as a subjective, perceptual belief construct rather than on demonstrated mathematical creativity performance. Empirical evidence suggests that creative self-efficacy and critical thinking dispositions are positively associated with problem-solving strategies and performance through both direct and indirect cognitive-affective pathways (Lee et al., 2026; Tasgin & Dilek, 2023). Recent empirical research further demonstrates the utility of structural equation modeling for examining complex relationships among mathematics-related problem-solving and 21st-century cognitive skills. For example, a recent study using structural equation modeling reported significant associations among project-based learning, critical thinking, problem-solving, and student engagement, with critical thinking and problem-solving also functioning as statistically significant mediating variables (Rehman et al., 2026). Such findings further support the value of theoretically specified pathway models for examining how interconnected cognitive and affective constructs are statistically associated within mathematics education contexts.

### 2.5. Theoretical Rationale for the Proposed Structural Sequence

Although the three constructs examined in this study are conceptually related, the proposed structural ordering was specified a priori on theoretical rather than statistical grounds. The model conceptualizes problem-solving self-efficacy as an antecedent motivational belief, critical thinking disposition as a cognitive disposition through which this motivational orientation may be expressed, and mathematical creativity self-efficacy as a more domain-specific efficacy belief associated with engagement in higher-order mathematical thinking. This conceptualization does not imply that these constructs are unidirectionally or temporally ordered in all contexts; rather, it provides a theoretically motivated basis for examining their proposed statistical relationships within the present cross-sectional design.

This sequence is primarily grounded in Social Cognitive Theory (Bandura, 1997), which conceptualizes self-efficacy beliefs as important motivational determinants of individuals’ willingness to initiate, sustain, and regulate cognitively demanding activities. Individuals who perceive themselves as capable of solving mathematical problems are more likely to invest effort, persist in the face of difficulty, and engage with challenging cognitive tasks rather than avoid them. Accordingly, problem-solving self-efficacy was conceptualized as an enabling motivational condition that may be associated with greater readiness to engage in reflective and analytical thinking during mathematical problem solving. Importantly, this theoretical rationale concerns the motivational function of efficacy beliefs and does not by itself establish that problem-solving self-efficacy temporally precedes critical thinking disposition.

The proposed intermediary role of critical thinking disposition is further informed by the Dual-Process Theory of reasoning (Evans & Stanovich, 2013). From this perspective, effective higher-order reasoning involves not only the cognitive capacity to perform demanding operations but also a willingness to engage in reflective, deliberate, and analytically demanding processing rather than relying exclusively on intuitive responses. Within this framework, critical thinking disposition represents an orientation toward engaging in reflective reasoning when individuals encounter complex problems. Individuals with stronger problem-solving self-efficacy may be more willing to invest the cognitive effort required for such reflective processing, thereby increasing the likelihood that critical thinking dispositions are expressed during mathematical problem solving. Thus, the proposed pathway from problem-solving self-efficacy to critical thinking disposition represents a theoretically grounded hypothesis concerning their structural association.

The final pathway linking critical thinking disposition to mathematical creativity self-efficacy is likewise theoretically motivated. Mathematical creativity requires not only the generation of novel ideas but also their evaluation, refinement, and justification. Individuals who habitually examine evidence, evaluate alternative solution strategies, and critically reflect on mathematical reasoning may have greater opportunities to refine and validate their own mathematical ideas. Such experiences are theoretically relevant to the development of efficacy beliefs because, according to Social Cognitive Theory (Bandura, 1997), successful performance experiences constitute a principal source of self-efficacy beliefs. Accordingly, critical thinking disposition was hypothesized to be statistically associated with mathematical creativity self-efficacy through opportunities for evaluating, refining, and validating original mathematical solutions. This rationale provides a theoretical basis for the proposed direction while recognizing that the reciprocal association between creative efficacy and critical engagement remains plausible.

Accordingly, the proposed structural sequence represents a theory-informed cognitive-affective framework in which motivational beliefs are associated with reflective cognitive engagement, which is in turn associated with domain-specific creative efficacy beliefs. The ordering is therefore intended as a theoretically motivated representation of the relationships among the constructs. Alternative structural orderings remain conceptually plausible, including models in which critical thinking disposition precedes both forms of self-efficacy or mathematical creativity self-efficacy is associated with subsequent critical engagement. The present framework therefore provides one theoretically grounded specification against which alternative directional models can be considered, rather than assuming that the observed cross-sectional associations uniquely determine the direction of the relationships.

Within mathematics teacher education, examining these associations is particularly relevant because problem-solving self-efficacy, critical thinking disposition, and mathematical creativity self-efficacy may jointly characterize how preservice teachers approach complex and open-ended mathematical activity. Rather than assuming that one construct necessarily produces another, the present study examines whether the proposed configuration provides an empirically consistent representation of the observed relationships. Specifically, the study focuses on both the direct statistical association between problem-solving self-efficacy and mathematical creativity self-efficacy and the indirect statistical association through critical thinking disposition.

## 3. Method

### 3.1. Research Design

**Figure 1:**
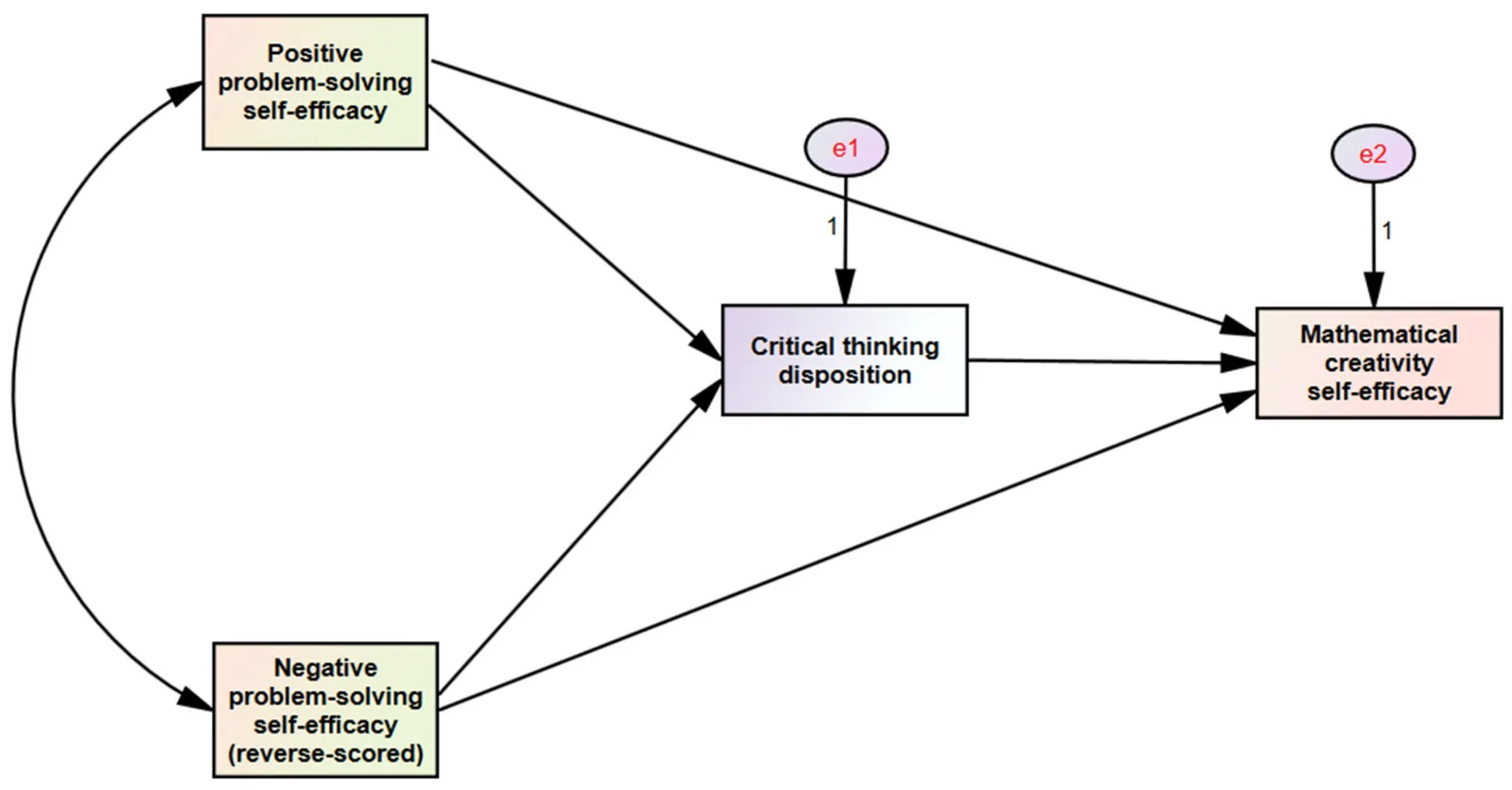
Hypothesized mediation path model for mathematical creativity self-efficacy.

This study simultaneously examines the direct association between problem-solving self-efficacy and mathematical creativity self-efficacy, as well as the indirect statistical association through critical thinking disposition, using observed-variable path analysis within a structural equation modeling framework (Byrne, 2016; Kline, 2023). Specifically, path analysis based on composite scores was conducted to examine the hypothesized relationships among the study variables. In the proposed model, the positive and negative dimensions of problem-solving self-efficacy are specified as exogenous variables, critical thinking disposition functions as the mediating variable, and mathematical creativity self-efficacy is specified as the endogenous dependent variable. Grounded in the conceptual framework outlined above, the model statistically examines whether the association between problem-solving self-efficacy and mathematical creativity self-efficacy is represented through both direct and indirect pathways (Figure 1). Importantly, the specified pathways represent theoretically motivated associations rather than empirically established causal relationships, given the cross-sectional nature of the data.

### 3.2. Research Sample

The study was conducted within the teacher education system in Turkey, and data were collected over a four-month period between December 2025 and March 2026. The target population was defined according to specific inclusion criteria: participants were required to be actively enrolled in accredited, four-year undergraduate elementary mathematics teacher education programs at public universities. Using a convenience sampling strategy, a total of 600 paper-based survey packages were distributed across five major public universities. Of these, 554 fully completed and valid responses were obtained, corresponding to a final response rate of 92.3%. The inclusion of participants from multiple institutions provided a diversified empirical basis for examining the relationships among the study variables within the specified population (Creswell & Creswell, 2023).

The institutional distribution of the participants across the five anonymized public universities was as follows: University A (*n* = 63, 11.4%), University B (*n* = 98, 17.7%), University C (*n* = 102, 18.4%), University D (*n* = 108, 19.5%), and University E (*n* = 183, 33.0%). Thus, although the participants were drawn from the same general teacher education context, the sample represented five different institutional settings. The potential implications of this institutional structure for the statistical analyses were examined separately as part of the data analysis procedures described in Section 3.7.

From a demographic perspective, 79% of the participants were female (*n* = 440) and 21% were male (*n* = 114). The distribution across grade levels was 10% (*n* = 55) first-year, 16% (*n* = 89) second-year, 39% (*n* = 217) third-year, and 35% (*n* = 193) fourth-year students. The final sample size (*N* = 554) substantially exceeded the commonly recommended minimum sample-size requirements for the reliable estimation of path model parameters and provided adequate statistical power for the planned analyses (Kline, 2023).

### 3.3. Data Collection Instruments

In order to measure the variables included in the path model, three previously developed and validated measurement instruments were employed in the study. The psychometric indicators reported in this section are drawn from the original scale development or adaptation studies. Psychometric results recalculated using the current study sample are reported separately in Section 3.4 and Table 1.

***Problem-Solving Self-Efficacy Scale***: The 15-item scale developed by Kıral-Demir and Katrancı (2023) comprises two empirically distinct subdimensions: positive problem-solving self-efficacy and negative problem-solving self-efficacy (reverse-scored). The instrument is structured as a 5-point Likert-type scale. The negative problem-solving self-efficacy (reverse-scored) subscale corresponds to the “negative self-efficacy for problem-solving” dimension in the original terminology and consists of negatively worded items (e.g., “I generally feel inadequate when solving mathematics problems,” “I have difficulty solving mathematics problems because I usually cannot understand them,” and “Compared with my classmates, I consider myself less competent at solving mathematics problems.”). Prior to computing composite scores, all negatively worded items were reverse-scored. Consequently, higher scores on both subdimensions indicate higher levels of perceived problem-solving self-efficacy. Thus, the revised terminology preserves the original two-factor structure of the instrument while making clear that the term negative refers to the wording of the original items rather than to the direction of the scores used in the analyses. In the original scale development study, Confirmatory Factor Analysis (CFA) results (χ^2^/df = 1.84; RMSEA = 0.06; CFI = 0.98) indicated good model fit. Cronbach’s alpha coefficients were reported as 0.91 for the overall scale and 0.88 and 0.87 for the two subdimensions, respectively.

***UF/EMI Critical Thinking Disposition Scale***: Adapted by Ertaş Kılıç and Şen (2014), this 25-item scale measures three dimensions: engagement, cognitive maturity, and innovativeness. In the Turkish adaptation study, the reported validity analyses (χ^2^/df = 2.85; RMSEA = 0.07; CFI = 0.94) indicated acceptable model fit. The reported internal consistency coefficient was 0.91 for the total scale, while coefficients for the subdimensions ranged from 0.67 to 0.88.

***Mathematical Creativity Self-Efficacy Perception Scale***: Developed by Açıkgül and Aksungur Altun (2022), this 27-item scale represents the dimensions of fluency, flexibility, and originality. In the original scale development study, the factor structure explained 64.03% of the total variance, and the reported CFA findings (χ^2^/df = 2.46; RMSEA = 0.071; CFI = 0.99; SRMR = 0.042) indicated excellent model-data fit. The reported Cronbach’s alpha coefficients were 0.97 for the total scale and ranged from 0.93 to 0.95 for the subdimensions.

### 3.4. Internal Consistency and Construct Validity Analysis

The construct validity of the measurement instruments was examined using Confirmatory Factor Analysis (CFA) with maximum likelihood estimation. Model fit was evaluated using χ^2^/df, RMSEA, CFI, GFI, and RMR, and Cronbach’s alpha (α) coefficients were calculated to assess the internal consistency of the scales and their respective subdimensions. The results are presented in Table 1.

The Problem-Solving Self-Efficacy Scale demonstrated good fit within its correlated two-factor structure (χ^2^/df = 2.48, RMSEA = 0.05, CFI = 0.95, GFI = 0.95, RMR = 0.03). The internal consistency coefficients for its two subdimensions were 0.81 and 0.84, respectively, indicating satisfactory reliability.

Given the multidimensional conceptualization of critical thinking disposition, three measurement representations of the Critical Thinking Disposition Scale were examined. First, a one-factor model in which all items loaded on a single general critical thinking disposition factor yielded χ^2^/df = 3.58, RMSEA = 0.07, CFI = 0.84, GFI = 0.87, and RMR = 0.04. The overall scale demonstrated high internal consistency (Cronbach’s α = 0.90). Second, a second-order model comprising three first-order factors (engagement, cognitive maturity, and innovativeness) under a higher-order critical thinking disposition factor produced χ^2^/df = 3.95, RMSEA = 0.07, CFI = 0.82, GFI = 0.86, and RMR = 0.06, with an overall reliability coefficient of α = 0.90. Third, the correlated three-factor model, in which engagement, cognitive maturity, and innovativeness were specified as distinct but correlated dimensions, yielded χ^2^/df = 4.01, RMSEA = 0.07, CFI = 0.81, GFI = 0.86, and RMR = 0.06. The corresponding subdimension reliability coefficients were 0.85, 0.70, and 0.70, respectively. Therefore, the alternative CFA specifications did not provide uniformly strong evidence for a single preferred measurement representation of critical thinking disposition. Although the RMSEA values were within an acceptable range across the three specifications, the CFI and GFI values remained below commonly recommended benchmarks, indicating that the measurement structure of the scale should be interpreted cautiously (Hu & Bentler, 1999; Kline, 2023). Importantly, the use of an overall critical thinking disposition composite score in the subsequent observed-variable path analyses was not based on an assumption that the multidimensional CFA specifications produced poorer fit. Rather, the composite score was retained because the primary research questions and hypothesized structural models concerned critical thinking disposition as an overall disposition construct, and the analytical framework employed observed scale scores rather than latent multidimensional factors. Thus, the decision was based on conceptual alignment with the study objectives and the observed-variable path analytic framework.

To further evaluate the psychometric properties of the overall critical thinking disposition score, Composite Reliability (CR) and Average Variance Extracted (AVE) were calculated. Composite Reliability was satisfactory (CR = 0.904), whereas the AVE value (0.273) was below the recommended threshold, indicating limited convergent validity. Although satisfactory composite reliability may partially compensate for a low AVE (Fornell & Larcker, 1981; Malhotra & Dash, 2011), the low AVE, together with the CFA results, warrants caution when interpreting critical thinking disposition as a homogeneous overall construct. These measurement limitations are therefore explicitly considered when interpreting the structural findings.

The Mathematical Creativity Self-Efficacy Scale was examined using both a correlated three-factor model comprising fluency, flexibility, and originality and a second-order hierarchical model. The two specifications produced statistically identical good fit indices (χ^2^/df = 2.38, RMSEA = 0.05, CFI = 0.94, GFI = 0.90, RMR = 0.03). The Cronbach’s alpha coefficients were 0.88 for fluency, 0.88 for flexibility, and 0.91 for originality, while the overall reliability coefficient for the second-order representation was 0.94. Taken together, the measurement analyses provide support for the reliability of the instruments while also indicating that the Critical Thinking Disposition Scale should be interpreted with appropriate caution, particularly with respect to convergent validity (Hu & Bentler, 1999; Kline, 2023; Tabachnick & Fidell, 2019).

### 3.5. Data Screening and Preliminary Analyses

Prior to the main analyses, the dataset was systematically screened to evaluate its suitability for path analysis. Of the 600 paper-based questionnaires distributed, 554 questionnaires met the study criteria and were retained for analysis, yielding a final response rate of 92.3%. At the item level, missing data were minimal, with only 45 missing responses across the 37,118 possible responses to the 67 scale items (0.12%). Given this negligible proportion of missing data, missing values were estimated using the Expectation-Maximization (EM) algorithm implemented in the Missing Value Analysis procedure in IBM SPSS Statistics Version 24 prior to the main analyses. Duplicate responses were also examined by comparing demographic information and response patterns, and no duplicate cases were identified. Response patterns were visually inspected to identify potential careless or inconsistent responding, and no questionnaires met the criteria for exclusion on this basis. Multivariate outliers were evaluated using Mahalanobis distance (D^2^) computed in IBM SPSS Amos Version 24 (IBM Corp., Armonk, NY, USA). Although a small number of observations exceeded the conventional significance criterion (*p*1 < 0.001 and *p*2 < 0.001), these cases were retained because they represented plausible observations rather than data-entry errors or invalid responses. Consistent with Byrne (2016), retaining such observations preserves the natural variability of the target population.

### 3.6. Assessment of Common Method Bias

Because all study variables were measured using self-report questionnaires administered during the same data collection process, the potential influence of common method bias (CMB) was evaluated prior to the main analyses. As an initial assessment, Harman’s single-factor test was conducted using Principal Axis Factoring (Harman, 1976; Podsakoff et al., 2003). All measurement items were entered simultaneously into an unrotated exploratory factor analysis. According to the initial eigenvalues, the first factor accounted for 27.11% of the total variance, whereas the extraction sum of squared loadings indicated that the extracted first factor accounted for 26.09% of the variance. Both values were well below the commonly referenced 50% criterion, suggesting that a single factor did not account for the majority of covariance among the measurement items. Because Harman’s single-factor test alone has recognized limitations, an additional sensitivity analysis was conducted using the Common Latent Factor (CLF) approach within the confirmatory factor analysis framework (Podsakoff et al., 2003; Eichhorn, 2014). All observed indicators were specified to load both on their theoretical latent constructs and on a common latent factor representing shared method variance. The average variance attributable to the common latent factor, calculated from the squared standardized factor loadings, was 12.4%, indicating that common method variance was limited and unlikely to materially affect the estimated relationships among the study variables. Accordingly, the Common Latent Factor was used exclusively as a diagnostic sensitivity analysis. The final path analysis was estimated using the theoretical model without the inclusion of the common latent factor.

### 3.7. Data Analysis

Prior to conducting the path analysis, the assumptions of univariate and multivariate normality required for Maximum Likelihood (ML) estimation were examined (Table 2).

Analyses indicated that the skewness and kurtosis critical ratios of the problem-solving self-efficacy subdimensions exceeded ±1.96, and Mardia’s multivariate kurtosis coefficient (C.R. = 14.85) indicated a violation of multivariate normality. Accordingly, robust parameter estimation was performed using the Bias-Corrected (BC) bootstrap procedure with 5000 resamples implemented in IBM SPSS AMOS (Version 24). The bootstrap results yielded negligible bias values (|bias| ≤ 0.001), low standard errors (SE = 0.025–0.043), and relatively narrow confidence intervals, supporting the stability of the parameter estimates under non-normal data conditions (Nevitt & Hancock, 2001).

Within the study, descriptive statistics and Pearson correlation coefficients were first calculated, followed by observed-variable path analysis within the structural equation modeling framework to examine the direct and indirect associations among the variables. Specifically, the direct association between problem-solving self-efficacy and mathematical creativity self-efficacy and the indirect statistical association through critical thinking disposition were examined. Model evaluation included explained variance (R^2^), 95% bootstrap confidence intervals, and effect sizes. Analyses were conducted using AMOS. Indirect associations were estimated using a 5000-resample bootstrap procedure with 95% confidence intervals (CI). Effect sizes were interpreted based on Cohen (2013). The statistical significance of path coefficients was assessed using the critical ratio criterion (|C.R.| > 1.96) (Byrne, 2016). Model fit was evaluated using χ^2^/df, the absolute fit index GFI, the incremental/comparative fit indices TLI and CFI, and the error-based indices RMSEA and RMR (Hu & Bentler, 1999; Kline, 2023). Additional analyses examined the subdimensions of mathematical creativity self-efficacy. In the primary path models, critical thinking disposition was represented by an overall composite score to maintain conceptual alignment with the study objectives and the observed-variable path analytic framework. Maximum likelihood estimation and a 5000-resample bias-corrected (BC) bootstrap procedure were used to estimate direct, indirect, and total associations.

Because participants were recruited from five universities, potential clustering by institution was examined before estimating the structural models. Unconditional random-intercept models were initially estimated in IBM SPSS Statistics Version 24, yielding intraclass correlation coefficients of ICC = 0.0096 for critical thinking disposition and ICC = 0.0141 for mathematical creativity self-efficacy. Although these ICC values were small, the magnitude of an ICC should be considered together with cluster size when evaluating the potential consequences of clustered data (Hox et al., 2017; Muthén & Satorra, 1995). Given an average cluster size of approximately 111 participants per university, the corresponding design effects were approximately 2.05 for critical thinking disposition and 2.55 for mathematical creativity self-efficacy. Because design effects around or above 2.0 may indicate a non-negligible influence of clustering on sampling variance (Muthén & Satorra, 1995), and because ICC estimates based on only five clusters may be relatively unstable, an additional fixed-effects sensitivity analysis was conducted. Specifically, both the base and detailed path models were re-estimated after including four university dummy variables as covariates, with one university serving as the reference category.

The focal standardized path estimates remained highly stable after adjustment for university membership. In the Base Model, the standardized coefficient from positive problem-solving self-efficacy to critical thinking disposition remained essentially unchanged at β = 0.64. The coefficient from positive problem-solving self-efficacy to mathematical creativity self-efficacy changed only slightly from β = 0.17 to β = 0.19, while the coefficient from critical thinking disposition to mathematical creativity self-efficacy changed from β = 0.48 to β = 0.46. The coefficient from the negative problem-solving self-efficacy (reverse-scored) dimension to mathematical creativity self-efficacy changed only slightly from β = 0.15 to β = 0.16. Overall, the largest absolute change among the focal standardized coefficients in the Base Model was approximately 0.02, and all focal paths remained statistically significant (*p* ≤ 0.001).

A similarly stable pattern was observed in the Detailed Model. After adjustment for university fixed effects, the standardized coefficient from positive problem-solving self-efficacy to critical thinking disposition remained essentially unchanged at β = 0.64, as did the coefficient from positive problem-solving self-efficacy to fluency at β = 0.11. The coefficient from positive problem-solving self-efficacy to flexibility also remained unchanged at β = 0.21, whereas the coefficient from the negative problem-solving self-efficacy (reverse-scored) dimension to originality changed from β = 0.11 to β = 0.12. Among the paths from critical thinking disposition to the mathematical creativity self-efficacy dimensions, the coefficient for originality changed from β = 0.46 to β = 0.44, the coefficient for flexibility remained essentially unchanged at β = 0.44, and the coefficient for fluency changed from β = 0.48 to β = 0.47. The coefficient from the negative problem-solving self-efficacy (reverse-scored) dimension to fluency remained essentially unchanged at β = 0.22, while its coefficient for flexibility remained β = 0.14. The largest absolute change across the focal standardized coefficients in the Detailed Model was approximately 0.02, with the focal paths retaining their statistical significance. Although some university dummy variables were individually associated with particular outcomes, controlling for university membership did not materially alter the magnitude, direction, or overall pattern of the focal structural relationships. Taken together, these sensitivity analyses suggest that the substantive conclusions of both path models are robust to adjustment for observed between-university differences.

## 4. Results

### 4.1. Descriptive Statistics for Problem-Solving Self-Efficacy, Critical Thinking Disposition, and Mathematical Creativity Self-Efficacy

The descriptive statistics for the study variables are presented in Table 3.

The positive subdimension of problem-solving self-efficacy (M = 4.15, SD = 0.48) reflected a high level of self-efficacy, whereas the negative subdimension (M = 3.96, SD = 0.67) indicated a moderate-to-high level. Critical thinking disposition (M = 3.90, SD = 0.44) and mathematical creativity self-efficacy (M = 3.67, SD = 0.54) likewise indicated above-average levels across the respective constructs. The concentration of scores within the moderate-to-high range, together with the relatively low standard deviations (0.44–0.67), indicates limited dispersion in participants’ responses and may suggest some degree of ceiling-related restriction in score variability. Such restriction can attenuate observed correlations and should therefore be considered when interpreting the magnitude of the bivariate and path associations.

### 4.2. Correlation Findings Among the Variables

The means, standard deviations, and full Pearson correlation matrix for the observed variables included in the Base and Detailed Models are presented in Table 4. Reporting the complete matrix provides the descriptive and bivariate information necessary for evaluating the relationships among the study variables and facilitates independent re-estimation of the observed-variable path models.

Positive problem-solving self-efficacy was positively associated with critical thinking disposition (r = 0.64, *p* < 0.01) and overall mathematical creativity self-efficacy (r = 0.54, *p* < 0.01). It was also positively associated with the mathematical creativity self-efficacy subdimensions of fluency (r = 0.51, *p* < 0.01), flexibility (r = 0.56, *p* < 0.01), and originality (r = 0.38, *p* < 0.01). Negative Problem-Solving Self-Efficacy (Reverse-Scored) showed comparatively weaker but statistically significant positive associations with critical thinking disposition (r = 0.25, *p* < 0.01), overall mathematical creativity self-efficacy (r = 0.34, *p* < 0.01), fluency (r = 0.38, *p* < 0.01), flexibility (r = 0.33, *p* < 0.01), and originality (r = 0.22, *p* < 0.01). The two problem-solving self-efficacy subdimensions were positively but relatively modestly correlated (r = 0.38, *p* < 0.01). Critical thinking disposition was strongly associated with overall mathematical creativity self-efficacy (r = 0.63, *p* < 0.01) and was also positively associated with fluency (r = 0.60, *p* < 0.01), flexibility (r = 0.60, *p* < 0.01), and originality (r = 0.49, *p* < 0.01). As expected, the overall mathematical creativity self-efficacy score was strongly correlated with its constituent subdimensions of fluency (r = 0.90, *p* < 0.01), flexibility (r = 0.88, *p* < 0.01), and originality (r = 0.89, *p* < 0.01). The three subdimensions were also positively interrelated, with correlations ranging from r = 0.64 to r = 0.75 (all *p*s < 0.01). Collectively, these bivariate findings indicate meaningful associations among the constructs examined in both the Base and Detailed Models. However, the correlations are interpreted as bivariate associations and do not, by themselves, establish the directional ordering specified in the proposed path models.

Because the overall mathematical creativity self-efficacy score is derived from its constituent subdimensions, the high correlations between the overall score and fluency, flexibility, and originality were expected. Importantly, the overall score and its subdimensions were not entered simultaneously as predictors or outcomes within the same structural model. Multicollinearity was therefore evaluated separately within the variable sets relevant to each stage of the path analyses. In the first stage, in which the two problem-solving self-efficacy dimensions were specified in relation to critical thinking disposition, the Variance Inflation Factor (VIF) values were 1.16 and the corresponding Tolerance values were 0.86. In the second stage, in which the problem-solving self-efficacy dimensions and critical thinking disposition were specified in relation to mathematical creativity self-efficacy, VIF values ranged from 1.19 to 1.72 and Tolerance values ranged from 0.58 to 0.84. Across both stages, all VIF values remained well below the conservative threshold of 3.0, and all Tolerance values exceeded the recommended minimum of 0.10 (Hair et al., 2019). Taken together, these diagnostics suggest that multicollinearity was unlikely to have materially affected the estimation or interpretation of the focal path coefficients.

### 4.3. Pathways, Standardized Direct, Indirect, and Total Effects, and Mediation Results Based on Bootstrap Confidence Intervals

**Figure 2:**
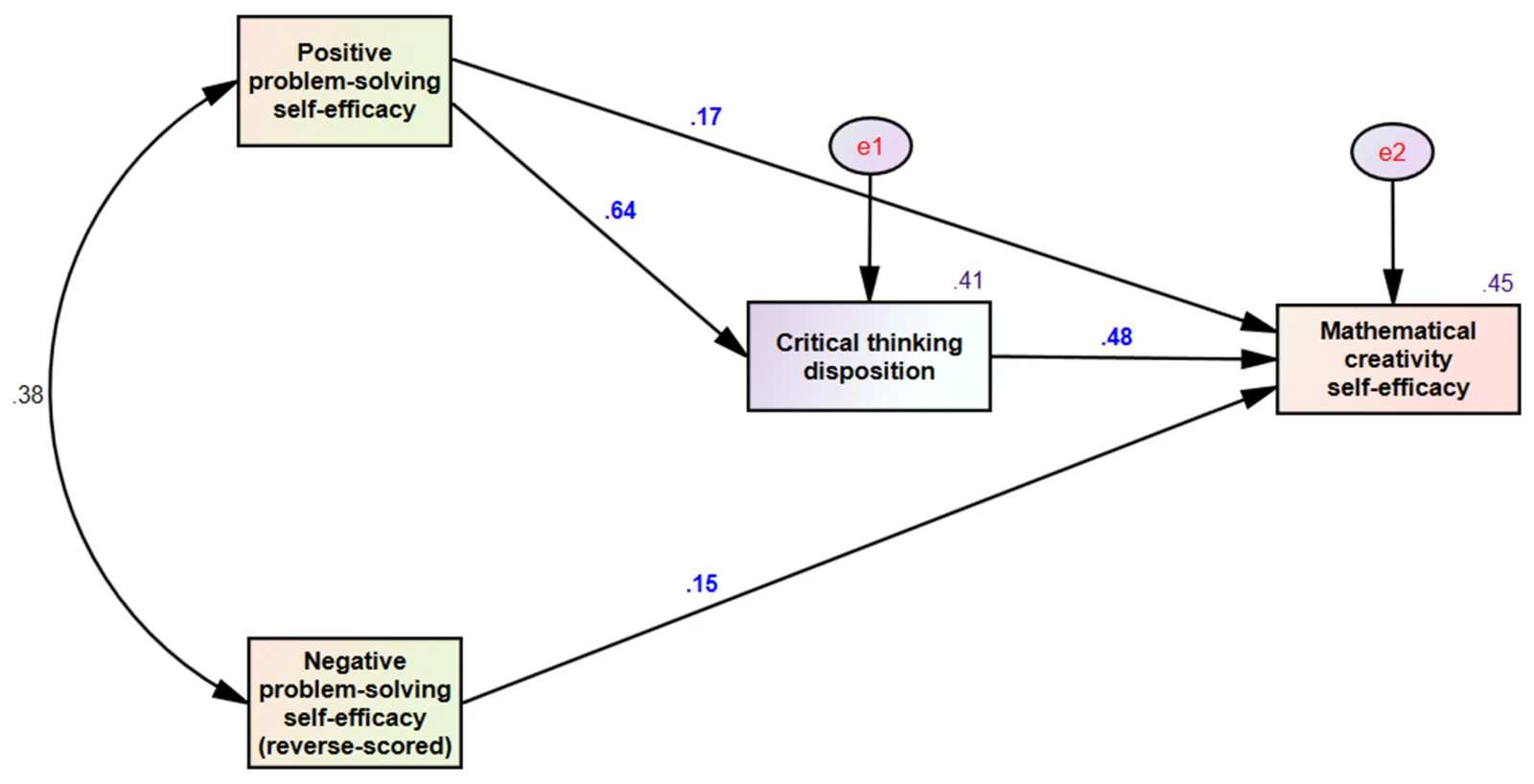
Path model illustrating the associations of positive and reverse-scored negative problem-solving self-efficacy with mathematical creativity self-efficacy through critical thinking disposition. *Note.* All path coefficients in the model were statistically different from zero at the 0.01 level (two-tailed).

The final parsimonious version of the proposed path model is presented in Figure 2.

The standardized direct, indirect, and total effect coefficients, together with their 95% confidence intervals (CI), are presented in Table 5. The bootstrap analysis (*N* = 5000) indicated that all retained path coefficients were statistically significant (*p* < 0.01), and none of the corresponding confidence intervals included zero.

Positive problem-solving self-efficacy was positively associated with critical thinking disposition (β = 0.64, *p* = 0.001, 95% CI [0.56, 0.70]). In turn, critical thinking disposition was positively associated with mathematical creativity self-efficacy (β = 0.48, *p* < 0.001, 95% CI [0.39, 0.56]). The direct association between negative problem-solving self-efficacy (reverse-scored) and mathematical creativity self-efficacy was positive but small in magnitude (β = 0.15, *p* < 0.001, 95% CI [0.09, 0.23]). Although the two problem-solving self-efficacy subdimensions were positively correlated, the magnitude of this association was relatively modest (r = 0.38). Their associations with critical thinking disposition also differed substantially: positive problem-solving self-efficacy showed a strong association (β = 0.64), whereas the initially hypothesized path from negative problem-solving self-efficacy (reverse-scored) to critical thinking disposition was negligible and statistically non-significant (β = 0.007). This pattern may be consistent with wording-related method variance and suggests that the two item sets should not be assumed to function as fully interchangeable indicators of the same underlying belief without further measurement investigation. Mediation analysis indicated a statistically significant indirect association between positive problem-solving self-efficacy and mathematical creativity self-efficacy through critical thinking disposition (β = 0.31, *p* < 0.001, 95% CI [0.25, 0.37]). The direct association remained statistically significant (β = 0.17, *p* < 0.001, 95% CI [0.10, 0.26]), yielding a total effect of β = 0.48 (*p* < 0.001, 95% CI [0.40, 0.55]). Taken together, these results are consistent with a partial statistical mediation pattern. Given the cross-sectional design, however, the indirect effect is interpreted as an indirect statistical association rather than evidence that critical thinking disposition causally mediates the relationship between problem-solving self-efficacy and mathematical creativity self-efficacy.

### 4.4. Explained Variance (R^2^), Confidence Intervals (CI), and Effect Sizes for Endogenous Variables

To evaluate the explanatory power of the observed-variable path model, the R^2^ values for the endogenous variables and their corresponding 95% bootstrap confidence intervals (CI) were examined (Table 6).

According to the analysis results, the predictor variables explained 41% of the variance in critical thinking disposition (R^2^ = 0.41, *p* = 0.001, 95% CI [0.31, 0.49]). The explained variance for mathematical creativity self-efficacy was R^2^ = 0.45 (*p* = 0.001, 95% CI [0.38, 0.51]). In line with Cohen’s (2013) criteria, these values correspond to a large effect size for both variables. Thus, the model accounted for substantial proportions of variance in critical thinking disposition and mathematical creativity self-efficacy. These findings indicate substantial explanatory associations among problem-solving self-efficacy, critical thinking disposition, and mathematical creativity self-efficacy within the specified path model.

### 4.5. Overall Model Fit

To evaluate the correspondence between the proposed observed-variable path model and the observed data, overall goodness-of-fit indices were examined. The results indicated that the chi-square value was non-significant (χ^2^(1) = 0.091, *p* = 0.762), and the χ^2^/df ratio (0.091) was well below the recommended threshold (χ^2^/df ≤ 3), indicating close correspondence between the specified model and the observed covariance structure (Kline, 2023). Error-based and approximate fit indices likewise indicated minimal residual discrepancy. The RMSEA value was 0.000 (90% CI [0.000, 0.076], PCLOSE = 0.878), and the RMR value was 0.001. Incremental fit indices were also examined: CFI (1.000), TLI (1.008), IFI (1.001), and NFI (1.000) all exceeded the critical threshold of 0.95 (Hu & Bentler, 1999). Furthermore, absolute fit indices (GFI = 1.000; AGFI = 0.999) indicated a very close correspondence between the specified structure and the observed data. Parsimony-adjusted indices (PNFI = 0.167; PCFI = 0.167) were also examined in evaluating the model’s relative parsimony. Additionally, the values of AIC (18.091), BIC (56.946), and ECVI (0.033) were lower than those of the independence model, while remaining close to the saturated model. Given the very low degrees of freedom of the model, however, these global fit indices and information criteria should be interpreted cautiously, as near-saturated models can exhibit excellent fit even when alternative theoretically plausible specifications may provide comparable representations of the observed covariance structure. Accordingly, the fit indices provide evidence of statistical correspondence between the specified pathways and the observed covariance structure.

### 4.6. Supplementary Analyses: Detailed Outcome Model

#### 4.6.1. Normality Assumption, Descriptive Statistics, and Correlations in the Detailed Outcome Model

To further elaborate on the integrative approach of the Base Model, a detailed outcome model was specified to examine mathematical creativity self-efficacy across its three dimensions: fluency, flexibility, and originality. In accordance with the principle of parsimony, critical thinking disposition was retained as a composite variable because multidimensional specifications produced suboptimal model fit.

Regarding the assessment of normality, some univariate deviations were observed. Notable departures from normality were detected in the positive and reverse-scored negative problem-solving self-efficacy subdimensions (skewness C.R. = −5.60; −5.42; kurtosis C.R. = 3.00; 2.43) and in the flexibility subdimension of creativity (skewness C.R. = −3.19; kurtosis C.R. = 3.66). For multivariate normality, Mardia’s kurtosis coefficient yielded a C.R. value of 12.73 (Base Model: 14.85). Although deviations from normality were observed, their magnitude did not warrant a change in the analytical strategy. Accordingly, parameter estimates were obtained using the Bias-Corrected (BC) Bootstrap method with 5000 resamples to provide robust confidence intervals.

The descriptive findings were broadly consistent with those of the Base Model. Among the creativity dimensions, flexibility demonstrated the highest mean score (M = 3.94, SD = 0.55), whereas originality showed relatively lower scores (M = 3.35, SD = 0.69). Pearson correlation analyses revealed significant positive associations among the variables (r = 0.22–0.75, *p* < 0.01). In particular, positive problem-solving self-efficacy showed strong associations with fluency and flexibility (r = 0.51–0.56), whereas critical thinking disposition demonstrated moderate-to-strong associations with all three creativity dimensions (r = 0.49–0.60). The relatively high intercorrelations among the creativity subdimensions (r = 0.64–0.75) support the interpretation that these dimensions are closely related while remaining empirically distinguishable. Taken together, this pattern of bivariate associations provides an empirical basis for examining the specified relationships within the detailed outcome model, while not in itself establishing their directional ordering.

#### 4.6.2. Pathways, Direct, Indirect, and Total Effects in the Detailed Outcome Model

**Figure 3:**
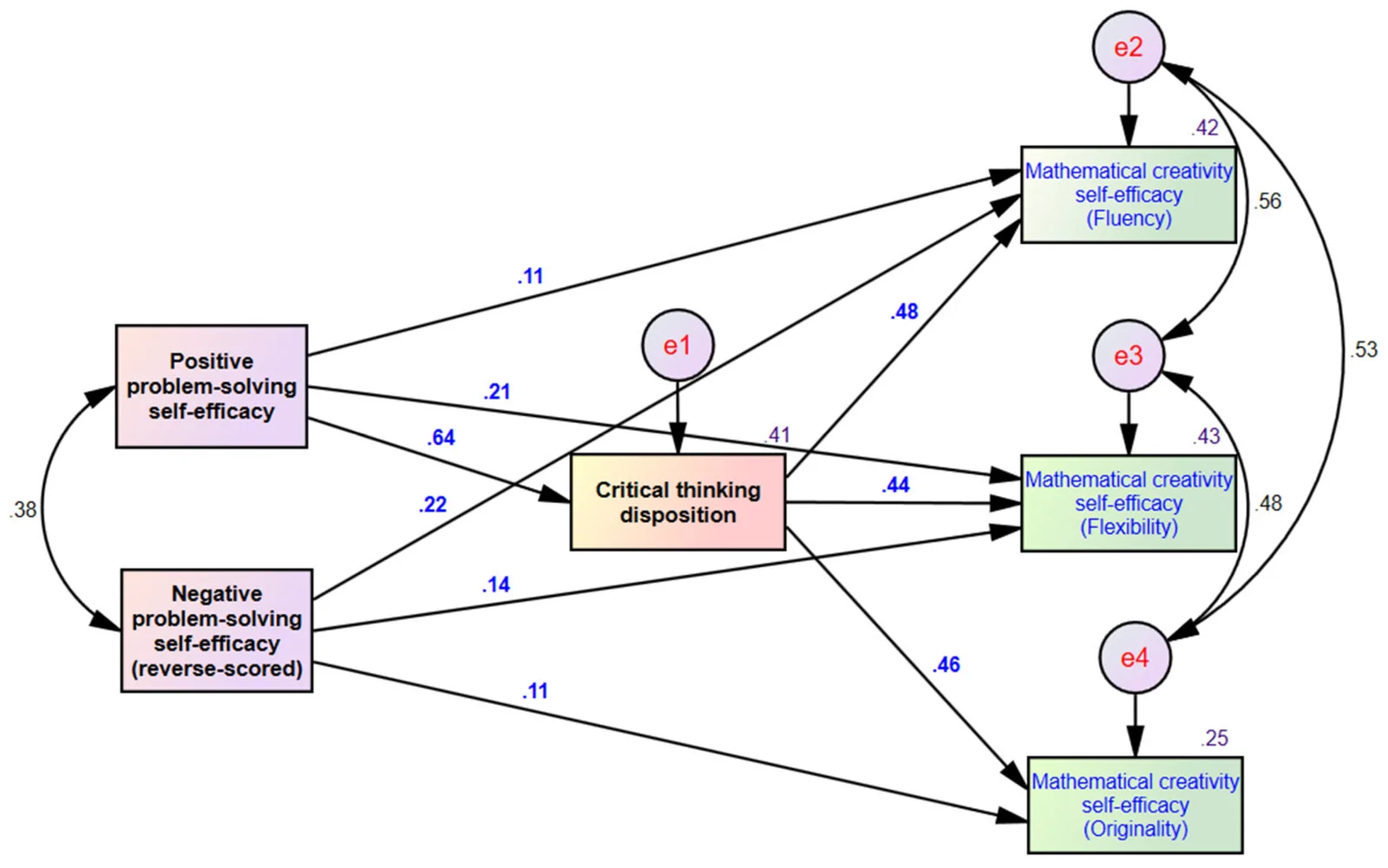
Detailed path model illustrating the associations of positive and reverse-scored negative problem-solving self-efficacy with mathematical creativity self-efficacy (fluency, flexibility, and originality) through critical thinking disposition. *Note.* All retained path coefficients in the model were statistically different from zero at the 0.01 level (two-tailed).

The final structure of the Detailed Outcome Model is presented in Figure 3.

In this model, the associations of positive and reverse-scored negative problem-solving self-efficacy with fluency, flexibility, and originality were examined with critical thinking disposition specified as a mediating variable. All parameter estimates were obtained using the BC bootstrap method with 5000 resamples and are reported with 95% confidence intervals (CI) (Table 7). The results indicated that the confidence intervals for all statistically significant effects excluded zero.

Path analysis showed that positive problem-solving self-efficacy was positively associated with critical thinking disposition (β = 0.64, 95% CI [0.56, 0.70], *p* = 0.001), representing a large effect size according to Cohen (2013). In contrast, negative self-efficacy did not demonstrate a significant direct association with critical thinking disposition and was associated with the dimensions of mathematical creativity self-efficacy primarily through direct pathways. Critical thinking disposition demonstrated significant positive associations with originality (β = 0.46, 95% CI [0.39, 0.53]), flexibility (β = 0.44, 95% CI [0.35, 0.51]), and fluency (β = 0.48, 95% CI [0.40, 0.55]) (all *p*s < 0.01). Mediation analysis indicated a statistically significant indirect association between positive problem-solving self-efficacy and originality through critical thinking disposition (indirect effect β = 0.29, 95% CI [0.24, 0.35], *p* < 0.001), while the corresponding direct pathway was not retained in the final parsimonious model. This pattern is consistent with a full statistical mediation configuration; however, given the cross-sectional design, it should not be interpreted as evidence of temporal or causal mediation.

For the flexibility and fluency dimensions, critical thinking disposition functioned as a partial statistical mediator. For flexibility, both the direct effect (β = 0.21, 95% CI [0.14, 0.30]) and indirect effect (β = 0.28, 95% CI [0.22, 0.34]) were statistically significant, producing a total effect of β = 0.49 (95% CI [0.42, 0.56]). Similarly, for fluency, the direct effect (β = 0.11, 95% CI [0.03, 0.17]) and indirect effect (β = 0.31, 95% CI [0.25, 0.37]) were statistically significant, yielding a total effect of β = 0.42 (95% CI [0.34, 0.48]). Overall, the findings indicate that the positive association between problem-solving self-efficacy and the dimensions of mathematical creativity self-efficacy was statistically accounted for, in part, by critical thinking disposition. The statistically significant direct, indirect, and total effects, together with bootstrap confidence intervals that excluded zero, provide evidence of stable statistical associations within the specified path model. These findings are interpreted as evidence of an empirically supported association structure rather than as evidence of causal mechanisms.

#### 4.6.3. Explained Variance (R^2^), Confidence Intervals (CI), and Effect Sizes for Endogenous Variables in the Detailed Outcome Model

To evaluate the explanatory power of the Detailed Outcome Model, the explained variance (R^2^), 95% bias-corrected bootstrap confidence intervals (CI), and effect sizes were calculated for the endogenous variables (Table 8).

The results showed that the problem-solving self-efficacy variables explained 41% of the variance in critical thinking disposition (R^2^ = 0.41, 95% CI [0.31, 0.49], *p* = 0.001), corresponding to a large effect size according to Cohen (2013). For the subdimensions of mathematical creativity self-efficacy, the explained variance for originality was R^2^ = 0.25 (95% CI [0.18, 0.31], *p* = 0.001), indicating a moderate effect. In contrast, flexibility (R^2^ = 0.43, 95% CI [0.35, 0.49], *p* = 0.001) and fluency (R^2^ = 0.42, 95% CI [0.35, 0.48], *p* = 0.001) demonstrated large effect sizes. These results indicate that critical thinking disposition and problem-solving self-efficacy account for substantial proportions of variance in the dimensions of mathematical creativity self-efficacy. The relatively narrow bootstrap confidence intervals further indicate that the estimated explained-variance values were reasonably stable within the present sample.

#### 4.6.4. Overall Model Fit of the Detailed Outcome Model

The fit of the Detailed Outcome Model was evaluated using multiple complementary fit indices. The chi-square statistic indicated a good fit between the model and the observed data (χ^2^ = 3.099, df = 2, *p* = 0.212). The χ^2^/df ratio of 1.55, which falls below the recommended threshold (χ^2^/df ≤ 3), further supports the model’s good fit (Kline, 2023). Regarding incremental fit indices, all values exceeded the recommended cutoff of 0.95: NFI = 0.998, IFI = 0.999, TLI = 0.995, and CFI = 0.999. Similarly, the absolute fit indices, GFI = 0.998 and AGFI = 0.980, were above acceptable thresholds. These results indicate a close correspondence between the specified path structure and the observed covariance structure (Hu & Bentler, 1999; Schermelleh-Engel et al., 2003).

Error-based indices further supported the adequacy of the model. The RMSEA value of 0.032 indicates a close approximation to the observed data. The corresponding 90% confidence interval for RMSEA [0.000, 0.096] and PCLOSE value of 0.586 provide additional support for acceptable population-level fit. Additionally, the RMR value of 0.004 indicates minimal discrepancies between the observed and estimated covariance matrices. Parsimony-based indices also supported the model’s adequacy. The values of AIC (41.099), BIC (123.125), and ECVI (0.074) were close to those of the saturated model and lower than those of the independence model, indicating that the model provides a relatively parsimonious representation of the observed data.

Taken together, the absolute, incremental, error-based, and parsimony-based indices indicate that the Detailed Outcome Model demonstrates good empirical fit with the observed data (Browne & Cudeck, 1993; Hu & Bentler, 1999; Kline, 2023; Meyers et al., 2017; Tabachnick & Fidell, 2019). These results support the adequacy of modeling the multidimensional structure of mathematical creativity self-efficacy in relation to critical thinking disposition and problem-solving self-efficacy.

#### 4.6.5. Sensitivity Analysis for Potential Content Overlap in Critical Thinking Disposition

To examine whether the association between critical thinking disposition and mathematical creativity self-efficacy was disproportionately influenced by conceptual overlap with the innovativeness subdimension, two complementary sensitivity analyses were conducted. First, mathematical creativity self-efficacy was positively associated with each of the three critical thinking disposition subdimensions: engagement (r = 0.64, *p* < 0.01), cognitive maturity (r = 0.47, *p* < 0.01), and innovativeness (r = 0.54, *p* < 0.01). Although innovativeness showed a moderately strong association with mathematical creativity self-efficacy, it was not the most strongly associated subdimension, as the correlation with engagement was larger.

As a further sensitivity check, both structural models were re-estimated using a critical thinking disposition composite from which the innovativeness items were excluded. In the Base Model, the standardized coefficient from positive problem-solving self-efficacy to critical thinking disposition remained essentially unchanged at β = 0.64. The coefficient from positive problem-solving self-efficacy to mathematical creativity self-efficacy remained essentially unchanged at β = 0.17, while the coefficient from critical thinking disposition to mathematical creativity self-efficacy changed slightly from β = 0.48 to β = 0.47. The coefficient from the negative problem-solving self-efficacy (reverse-scored) dimension to mathematical creativity self-efficacy remained essentially unchanged at β = 0.15. All four focal paths remained statistically significant (*p* < 0.001).

A similarly stable pattern was observed in the Detailed Model. After excluding the innovativeness items, the standardized coefficient from positive problem-solving self-efficacy to critical thinking disposition remained essentially unchanged at β = 0.64. The coefficient from positive problem-solving self-efficacy to fluency changed slightly from β = 0.11 to β = 0.10, whereas the coefficient from positive problem-solving self-efficacy to flexibility remained essentially unchanged at β = 0.21. The coefficient from the negative problem-solving self-efficacy (reverse-scored) dimension to originality also remained essentially unchanged at β = 0.11. Among the paths from critical thinking disposition to the mathematical creativity self-efficacy dimensions, the coefficient for originality changed slightly from β = 0.46 to β = 0.45, the coefficient for flexibility remained essentially unchanged at β = 0.44, and the coefficient for fluency changed slightly from β = 0.48 to β = 0.49. The coefficients from the negative problem-solving self-efficacy (reverse-scored) dimension to fluency and flexibility remained essentially unchanged at β = 0.22 and β = 0.14, respectively. All focal paths retained their statistical significance.

Taken together, the subdimension-level correlations and the re-estimated structural models indicate that excluding the innovativeness items produced only minimal changes in the focal path estimates. Thus, although conceptual overlap between innovativeness and mathematical creativity self-efficacy is plausible and should be acknowledged, the observed relationship between critical thinking disposition and mathematical creativity self-efficacy does not appear to be driven solely by the innovativeness component.

#### 4.6.6. Evaluation of Data-Informed Model Trimming via Nested Model Comparisons

To enhance model parsimony while maintaining transparency regarding the specification process, the initially hypothesized mediation models were evaluated through formal nested model comparisons. The complete theoretically hypothesized models were estimated first, after which statistically non-significant direct effects identified in these models were considered for constraint to zero. The resulting nested comparisons were used to determine whether imposing these constraints produced a statistically meaningful deterioration in overall model fit. Accordingly, this procedure is treated as a data-informed model trimming process rather than as an exclusively a priori specification decision (Byrne, 2016; Kline, 2023).

The comparison results are presented in Table 9 and Table 10. Table 9 reports the parameter estimates for the direct paths specified in the initially hypothesized structural models that were subsequently constrained to zero in the final parsimonious models. Table 10 summarizes the corresponding nested chi-square difference tests comparing the initially hypothesized models with the final parsimonious specifications.

Both initially hypothesized models represented saturated structural specifications (df = 0) and therefore reproduced the observed covariance matrix exactly. Although saturated models necessarily yield perfect global fit, they provide no degrees of freedom for evaluating model parsimony or empirical testability. Consequently, the relevant question was not whether the saturated models fit the data better, which is mathematically inevitable, but whether constraining the statistically non-significant direct effects to zero resulted in a statistically meaningful loss of fit.

For the Base Model, the initially hypothesized direct path from negative problem-solving self-efficacy (reverse-scored) to critical thinking disposition was not statistically significant (β = 0.007, SE = 0.023, CR = 0.302, *p* = 0.762). After this parameter was constrained to zero, the nested model comparison indicated no statistically significant deterioration in model fit (Δχ^2^ = 0.091, Δdf = 1, *p* = 0.762). Because only a single parameter was constrained in this comparison, the chi-square difference test provides essentially the same inferential information as the significance test for the individual path. Therefore, the nested comparison is interpreted here as a formal evaluation of the fit consequences of imposing the constraint rather than as independent confirmation of the non-significant path estimate.

Similarly, in the Detailed Model, two initially hypothesized direct effects were not statistically significant: the path from negative problem-solving self-efficacy (reverse-scored) to critical thinking disposition (β = 0.007, SE = 0.023, CR = 0.302, *p* = 0.762) and the direct path from positive problem-solving self-efficacy to the originality dimension of mathematical creativity self-efficacy (β = 0.125, SE = 0.072, CR = 1.737, *p* = 0.082). These two parameters were subsequently constrained to zero, and the corresponding nested model comparison indicated that the joint constraints did not produce a statistically significant deterioration in model fit (Δχ^2^ = 3.099, Δdf = 2, *p* = 0.212).

Thus, the final parsimonious specifications were obtained through a transparent, data-informed trimming procedure. The complete theoretically hypothesized models were estimated first, all relevant initially specified direct paths were evaluated and reported in Table 9, and statistically non-significant paths were subsequently constrained to zero. The nested chi-square difference tests were then used to assess whether these constraints materially worsened overall model fit. The results indicated that the imposed constraints did not result in statistically significant deterioration in fit, while yielding more parsimonious representations of the observed relationships.

Accordingly, the final models should be interpreted as data-informed simplifications of the initially hypothesized structural specifications rather than as models derived entirely from a priori specification. The nested comparisons indicate that the identified non-significant direct paths could be constrained to zero without a statistically significant loss of global model fit. However, these findings do not establish the retained specifications as uniquely correct, nor do they rule out alternative theoretically plausible structural models. Rather, they indicate that the trimmed models provide empirically adequate and more parsimonious representations of the observed data within the theoretical framework examined in the present study.

#### 4.6.7. Comparison of the Proposed and Reverse-Direction Competing Models

Because the proposed Base Model (df = 1) and Detailed Model (df = 2) were characterized by very low degrees of freedom, their global goodness-of-fit indices should be interpreted with caution, as near-saturated models are expected to exhibit excellent fit and may provide limited discrimination among competing structural specifications (Kline, 2023). Accordingly, rather than relying exclusively on global fit indices, the proposed models were compared with theoretically plausible reverse-direction specifications to examine whether the observed covariance structure was also compatible with an alternative directional ordering.

Specifically, complete reverse-direction models were estimated for both the Base and Detailed structures by reversing the structural paths specified in the corresponding proposed models. To maintain comparable model structures, theoretically justified correlated residuals were specified where appropriate. In the proposed Detailed Model, correlated residuals were retained among the three mathematical creativity self-efficacy dimensions (fluency, flexibility, and originality), reflecting their shared higher-order construct. In the reverse-direction models, correlated residuals were specified between the positive and negative problem-solving self-efficacy (reverse-scored) dimensions after these variables became endogenous.

The reverse-direction models were estimated without subsequent trimming of statistically non-significant paths. This distinction is important because the proposed models had previously undergone data-informed trimming, as described in Section 4.6.5. Consequently, differences in the number of statistically significant paths across the proposed and reverse-direction specifications were not treated as a criterion for determining model superiority. The comparison instead focused primarily on the extent to which the alternative directional specifications provided comparably plausible representations of the observed data. The results are presented in Table 11.

As shown in Table 11, both the proposed and reverse-direction models yielded very similar global fit indices and information criteria. For the Base Model, both the proposed model (χ^2^(1) = 0.091, *p* = 0.762, CFI = 1.000, RMSEA = 0.000, AIC = 18.091) and the reverse-direction model (χ^2^(1) = 1.373, *p* = 0.241, CFI = 0.999, RMSEA = 0.026, AIC = 19.373) demonstrated excellent overall fit. All structural paths were statistically significant in both specifications. The corresponding ΔAIC of 1.28 falls below the value generally regarded as providing meaningful discrimination between competing models (Burnham & Anderson, 2002). Thus, the Base Model results do not provide a strong empirical basis for preferring one directional specification over the other, and the observed data are compatible with both directional orderings.

A similar pattern was observed for the Detailed Model. The proposed model (χ^2^(2) = 3.099, *p* = 0.212, CFI = 0.999, RMSEA = 0.032, AIC = 41.099) and the reverse-direction model (χ^2^(2) = 2.599, *p* = 0.273, CFI = 1.000, RMSEA = 0.023, AIC = 40.599) likewise exhibited similarly excellent global fit. The ΔAIC of 0.50 indicates that neither specification is clearly preferred on the basis of information criteria alone (Burnham & Anderson, 2002). Accordingly, the global fit statistics and information criteria provide little empirical basis for distinguishing between the two directional specifications.

Inspection of the structural parameter estimates nevertheless revealed differences in the pattern of individual path significance. Whereas all nine structural paths retained in the proposed Detailed Model were statistically significant (all *p*s < 0.01), four paths in the untrimmed reverse-direction specification were not statistically significant: mathematical creativity self-efficacy (originality) to critical thinking disposition (β = 0.071, 95% CI [−0.021, 0.160], *p* = 0.122); mathematical creativity self-efficacy (fluency) to positive problem-solving self-efficacy (β = 0.062, 95% CI [−0.028, 0.150], *p* = 0.169); mathematical creativity self-efficacy (originality) to negative problem-solving self-efficacy (reverse-scored) (β = −0.067, 95% CI [−0.179, 0.047], *p* = 0.261); and mathematical creativity self-efficacy (flexibility) to negative problem-solving self-efficacy (reverse-scored) (β = 0.123, 95% CI [−0.002, 0.238], *p* = 0.055). These differences are reported descriptively rather than as evidence that the proposed model is empirically superior, because the proposed Detailed Model had undergone data-informed trimming whereas the reverse-direction model had not been subjected to an equivalent pruning procedure. Consequently, the number of statistically significant paths cannot provide a balanced basis for model selection.

To further examine the stability of the proposed structural specifications, a split-half stability check was conducted using multi-group path analysis. The full dataset (*N* = 554) was randomly divided into two independent subsamples of equal size (*n* = 277 and *n* = 277). Structural stability was examined by constraining the structural path coefficients to equality across the two subsamples and comparing the constrained and unconstrained models. For the Base Model, the constrained model did not differ significantly from the unconstrained model (Δχ^2^ = 1.190, Δdf = 4, *p* = 0.880). Likewise, for the Detailed Model, imposing equality constraints on the structural paths resulted in a non-significant change in model fit (Δχ^2^ = 6.112, Δdf = 9, *p* = 0.729). These findings indicate that the estimated structural relationships did not differ significantly across the two randomly generated subsamples, providing evidence of split-half stability in the proposed path models. This analysis is interpreted as a stability check rather than as a formal calibration–validation procedure because the models were not developed in one subsample and subsequently tested in the other.

Taken together, the competing-model comparisons indicate that the proposed and reverse-direction specifications provide highly similar representations of the cross-sectional data. This pattern is consistent with the possibility of statistically equivalent or near-equivalent structural models in covariance-based analyses (MacCallum et al., 1993). Accordingly, the proposed directional ordering is retained on theoretical grounds rather than because the empirical fit indices uniquely favor that specification. The split-half analysis provides additional evidence that the estimated relationships in the proposed models are stable across randomly generated subsamples, but it does not resolve the directional ambiguity inherent in the competing models.

## 5. Discussion

The findings of this study indicate that the relationships among problem-solving self-efficacy (positive and negative subdimensions), critical thinking disposition, and mathematical creativity self-efficacy exhibit a multidimensional and component-specific pattern. Overall, positive problem-solving self-efficacy was strongly associated with critical thinking disposition, and critical thinking disposition was, in turn, significantly associated with the fluency, flexibility, and originality dimensions of mathematical creativity self-efficacy. Moreover, the association between positive problem-solving self-efficacy and mathematical creativity self-efficacy comprised both direct and indirect components through critical thinking disposition, whereas the association with originality was entirely indirect through critical thinking disposition, consistent with a full mediation pattern in the specified model. These findings contribute to a more differentiated understanding of how self-efficacy beliefs, critical thinking disposition, and domain-specific creative self-beliefs may be related within an integrated cognitive-affective framework.

The strong association between positive problem-solving self-efficacy and critical thinking disposition is broadly consistent with the assumptions of Social Cognitive Theory. According to self-efficacy theory, individuals’ beliefs about their capabilities are associated with their willingness to engage in cognitively demanding activities and with the use of cognitive and metacognitive strategies (Bandura, 1997). Within this framework, preservice teachers with stronger problem-solving self-efficacy may be more inclined to engage in reflective, analytical, and evaluative approaches when confronting complex problems. The observed association is also consistent with previous findings linking self-efficacy with critical thinking and problem-solving-related dispositions (Orakci, 2023; Tasgin & Dilek, 2023). Nevertheless, because the present data were collected at a single time point, the findings do not establish that problem-solving self-efficacy temporally precedes or causes critical thinking disposition.

The significant associations between critical thinking disposition and the fluency, flexibility, and originality dimensions of mathematical creativity self-efficacy further suggest that critical thinking disposition is closely related to multiple dimensions of creative self-perceptions. This pattern is compatible with the view that critical thinking involves deliberate examination, comparison, evaluation, and reconsideration of alternative possibilities. Such processes may be relevant to how individuals appraise their own capacity to generate and refine mathematical ideas (Çavuş et al., 2026). Importantly, however, the present study measured mathematical creativity self-efficacy rather than mathematical creativity or actual creative performance. Therefore, the observed associations should not be interpreted as evidence that critical thinking disposition enables participants to generate more creative ideas, produce more original mathematical solutions, or improve their mathematical creativity. Rather, the findings indicate that stronger critical thinking disposition is associated with stronger beliefs about one’s capability to engage in mathematical creativity.

The findings further indicate that the association between positive problem-solving self-efficacy and mathematical creativity self-efficacy comprises both direct and indirect components through critical thinking disposition, with the latter functioning as a partial mediator in the specified model. This pattern suggests that the relationship between general problem-solving efficacy beliefs and domain-specific mathematical creativity self-efficacy may involve an intermediate cognitive disposition rather than being adequately characterized solely as a direct association. From a Social Cognitive Theory perspective, stronger efficacy beliefs are associated with greater willingness to invest effort and engage in demanding cognitive activities (Bandura, 1997). Consistent with this perspective, the present findings suggest that positive problem-solving self-efficacy is statistically connected to mathematical creativity self-efficacy both independently and through its association with critical thinking disposition. Similar relationships between self-efficacy and higher-order cognitive engagement have also been reported in previous research (Herianto et al., 2024).

The finding concerning the negative problem-solving self-efficacy (reverse-scored) subscale requires particular caution in interpretation. Because the items in this subscale were negatively worded and reverse-scored prior to analysis, higher composite scores represent higher rather than lower levels of problem-solving self-efficacy. Accordingly, the positive associations observed between this subscale and mathematical creativity self-efficacy are consistent with the broader pattern in which stronger problem-solving self-efficacy is associated with stronger mathematical creativity self-efficacy. This interpretation is broadly compatible with Bandura’s (1997) proposition that stronger efficacy beliefs are associated with individuals’ engagement with challenging tasks. Nevertheless, because the subscale consists exclusively of negatively worded items, some of the observed variance may reflect wording effects rather than a substantively distinct psychological process. Moreover, the present findings do not provide sufficient evidence to conclude that lower perceived competence reduces cognitive inhibition, decreases evaluation anxiety, or creates productive cognitive tension. Such mechanisms were not measured in the present study and should therefore be regarded as potential explanations to be examined in future research rather than as interpretations established by the current findings.

The differentiation observed at the subdimension level in the Detailed Outcome Model is also noteworthy. For fluency and flexibility, positive problem-solving self-efficacy demonstrated both direct and indirect associations through critical thinking disposition, indicating that these dimensions of mathematical creativity self-efficacy may be related to problem-solving self-efficacy through multiple statistical pathways. In contrast, the association between positive problem-solving self-efficacy and originality was entirely indirect through critical thinking disposition, consistent with a full mediation pattern in the specified model. This difference may indicate that the association between problem-solving efficacy beliefs and beliefs about originality is more closely connected to critical thinking disposition than are the corresponding associations involving fluency and flexibility. However, this interpretation concerns differences in the structure of statistical associations and should not be understood as evidence that critical thinking disposition is uniquely responsible for the development of originality.

The explanatory power of the model, as reflected in the R^2^ values, further indicates differentiation across the dimensions of mathematical creativity self-efficacy. Critical thinking disposition and problem-solving self-efficacy accounted for larger proportions of variance in fluency and flexibility than in originality, suggesting that originality may be associated with additional factors not represented in the present model. This interpretation is particularly important because originality represents a specific dimension of mathematical creativity self-efficacy rather than observed originality in mathematical performance. This differentiation may also be understood in terms of the distinct cognitive demands associated with the three dimensions of mathematical creativity self-efficacy. Problem-solving self-efficacy and critical thinking disposition may be more strongly associated with fluency and flexibility because these dimensions emphasize the generation of multiple ideas and the use of varied solution strategies, processes that are central to mathematical creativity (Leikin, 2009; Silver, 1997). By contrast, originality requires the production of ideas that are not only different but also novel or uncommon, making it conceptually distinct from the generation of greater numbers or varieties of responses (Runco & Jaeger, 2012; Sriraman, 2005). From this perspective, critical thinking may have a more complex relationship with originality: although evaluative and convergent thinking are important for examining and refining novel ideas, they do not themselves guarantee the generation of novelty (Cropley, 2006). Indeed, creativity involves a dynamic interplay between the generation of new possibilities and their subsequent evaluation and development (Walia, 2019). The lower proportion of explained variance observed for originality, compared with fluency and flexibility, may therefore indicate that originality-related mathematical creativity self-efficacy is associated with additional factors beyond the problem-solving self-efficacy and critical thinking disposition examined in the present model. For example, prior research suggests that creative self-efficacy is associated with mastery- and performance-related beliefs and feedback concerning creative ability (Beghetto, 2006). Thus, the comparatively lower explanatory power for originality may reflect the multidimensional nature of creative self-beliefs and the possibility that originality is associated with additional cognitive, motivational, and contextual factors not represented in the present model.

## 6. Conclusions, Implications, and Research Directions

Overall, the findings indicate that mathematical creativity self-efficacy is statistically associated with both problem-solving self-efficacy and critical thinking disposition. By considering these constructs within a single observed-variable path model, the study provides a more integrated perspective on how efficacy beliefs and cognitive dispositions may be related to perceptions of mathematical creative competence. The findings suggest that the association between problem-solving self-efficacy and mathematical creativity self-efficacy is not adequately represented by a direct relationship alone, as critical thinking disposition accounts for part of this association and, in the case of originality, accounts for the entire indirect pathway in the specified model. This pattern is broadly consistent with 21st-century skills frameworks emphasizing the interrelated roles of confidence, cognitive engagement, and creative thinking (OECD, 2024; Vincent-Lancrin et al., 2019). However, because the present study examined self-reported perceptions rather than observed mathematical creativity or changes resulting from an intervention, these findings should not be interpreted as demonstrating that increasing self-efficacy or critical thinking will improve creative performance.

The findings nevertheless provide several implications for preservice mathematics teacher education. Rather than recommending the generic simultaneous development of self-efficacy and critical thinking, the component-specific pattern observed in the Detailed Outcome Model suggests that attention to critical thinking disposition may be particularly relevant to originality-related mathematical creativity self-efficacy. The association between positive problem-solving self-efficacy and originality was entirely indirect through critical thinking disposition, whereas the corresponding relationships with fluency and flexibility included both direct and indirect components. This distinction suggests that teacher education programs may benefit from explicitly incorporating opportunities for preservice teachers to examine alternative mathematical approaches, evaluate the adequacy of proposed solutions, and reflect on the reasoning underlying different solution strategies. Such practices may be particularly relevant when the instructional goal is to strengthen preservice teachers’ perceptions of their capability to generate original mathematical ideas. These implications should be regarded as theoretically informed possibilities rather than evidence-based prescriptions for intervention, because no instructional intervention or change in the study variables was examined in the present research.

Future research should further distinguish between mathematical creativity self-efficacy and actual mathematical creativity. Because the present study assessed beliefs about creative capability rather than creative performance, subsequent investigations should combine self-report measures of mathematical creativity self-efficacy with performance-based mathematical creativity tasks. Such designs would make it possible to examine whether the observed associations among problem-solving self-efficacy, critical thinking disposition, and mathematical creativity self-efficacy correspond to differences in actual creative performance. Longitudinal and intervention-based research could further determine whether changes in these constructs are associated with subsequent changes in creative performance.

Several limitations warrant cautious interpretation of the findings. First, the cross-sectional nature of the data precludes drawing definitive conclusions regarding causality or temporal ordering. Although the proposed structural model was derived from established theoretical frameworks, alternative directional specifications remain theoretically plausible. For example, stronger mathematical creativity self-efficacy may itself encourage greater engagement in critical thinking, or critical thinking disposition may precede the development of both efficacy beliefs. Similarly, reciprocal relationships may exist between problem-solving self-efficacy and mathematical creativity self-efficacy, whereby experiences of competence in one domain are associated with beliefs in another. Because all variables were measured at a single point in time, the present study cannot empirically distinguish among these competing temporal sequences. Accordingly, the observed mediation should be interpreted as an indirect statistical association within the specified path model rather than as evidence of a chronological causal sequence. The proposed model should therefore be understood as a theoretically preferred and empirically consistent representation of the observed relationships rather than as definitive evidence of causal ordering. Longitudinal, cross-lagged, and experimental research designs are needed to evaluate the relative plausibility of alternative directional models and to examine potential reciprocal relationships over time.

Second, although both Harman’s single-factor test and the Common Latent Factor sensitivity analysis suggested that common method variance was unlikely to have substantially biased the estimated relationships, all variables were measured using self-report questionnaires administered to the same participants at a single point in time. Consequently, residual influences of response styles, generalized self-confidence, and other sources of common method variance cannot be completely excluded. Future studies should therefore consider multimethod and longitudinal approaches that incorporate performance-based indicators, repeated measurements, and, where appropriate, data from additional sources. Such designs would provide a stronger basis for determining whether the observed associations extend beyond shared self-report measurement.

Another limitation concerns the operationalization of critical thinking disposition. For the purposes of the present study, the construct was represented by a single composite score, consistent with the observed-variable path analysis employed. Although the scale demonstrated high internal consistency, the relatively low AVE suggests that convergent validity should be interpreted with caution. Future studies employing latent-variable structural models may provide a more rigorous evaluation of the construct while explicitly accounting for measurement error. More broadly, the present study utilized scale and subscale composite scores as observed variables within a path analysis framework rather than estimating a full structural equation model with latent variables derived from individual items. Consequently, the analyzed models do not explicitly account for measurement error, which may affect the estimated parameter relationships. Future research should therefore consider full latent-variable structural equation modeling (SEM) to replicate and extend these findings while accounting for measurement error at the item level.

Finally, although the data were collected from five different public universities, the sample was obtained through convenience sampling, which limits the external validity and nationwide generalizability of the findings. The sample should therefore be understood as representing the participating preservice elementary mathematics teachers from the sampled Turkish public universities rather than the broader population of preservice mathematics teachers in Turkey. Statistical diagnostics indicated that institutional clustering was negligible; however, the absence of substantial clustering does not resolve the issue of population representativeness. Accordingly, the findings should be generalized beyond the participating institutions and sampling context with caution. Future studies employing stratified random sampling across a broader range of geographical regions and institutional types are needed to evaluate the broader applicability of the tested path model.

Taken together, the contribution of the present study lies in providing a theoretically motivated and empirically consistent account of the associations among problem-solving self-efficacy, critical thinking disposition, and mathematical creativity self-efficacy. The findings particularly highlight the differentiated pattern across originality, fluency, and flexibility and suggest that critical thinking disposition may be especially relevant to the statistical association between problem-solving self-efficacy and originality-related creative self-beliefs. At the same time, the study does not establish a unique causal sequence, demonstrate changes in the constructs, or provide evidence of improved mathematical creativity. Future longitudinal, cross-lagged, experimental, and multimethod research is therefore needed to determine whether the observed structural pattern reflects developmental processes and whether the proposed relationships extend to actual mathematical creative performance.

## Figures and Tables

**Table 1:** Confirmatory factor analysis and reliability results for the scales.

Analysis Model	Number of Factors	χ^2^/df	RMSEA	CFI	GFI	RMR	Cronbach’s α
Problem Solving Self-Efficacy Scale (PSSES) (Correlated Model)	2	2.48	0.05	0.95	0.95	0.03	(0.81; 0.84)
Critical Thinking Disposition Scale (CTDS) (Composite Model)	1	3.58	0.07	0.84	0.87	0.04	0.90
Critical Thinking Disposition Scale (CTDS) (Second-order Model)	3 + 1	3.95	0.07	0.82	0.86	0.06	0.90
Critical Thinking Disposition Scale (CTDS) (Correlated Model)	3	4.01	0.07	0.81	0. 86	0.06	(0.85; 0.70; 0.70)
Mathematical Creativity Self-Efficacy Scale (MCSES) (Correlated Model)	3	2.38	0.05	0.94	0.90	0.03	(0.88; 0.88; 0.91) 0.94

*Note.* For the Mathematical Creativity Self-Efficacy Scale (MCSES), the correlated factors model and the second-order (hierarchical) model produced statistically identical fit indices.

**Table 2:** Normality values of the variables.

Variables	Min	Max	Skewness	C.R.	Kurtosis	C.R.
Positive problem-solving self-efficacy	2.22	5.00	−0.58	−5.60	0.62	3.00
Negative problem-solving self-efficacy (reverse-scored)	1.17	5.00	−0.56	−5.42	0.51	2.43
Critical thinking disposition	2.27	5.00	−0.02	−0.19	0.20	0.97
Mathematical creativity self-efficacy	1.59	4.96	−0.19	−1.84	0.43	2.07
Multivariate					8.74	14.85

**Table 3:** Descriptive statistics for the study variables.

Variables	N	Min	Max	Mean	SD
Positive problem-solving self-efficacy	554	2.22	5.00	4.15	0.48
Negative problem-solving self-efficacy (reverse-scored)	554	1.17	5.00	3.96	0.67
Critical thinking disposition	554	2.27	5.00	3.90	0.44
Mathematical creativity self-efficacy	554	1.59	4.96	3.67	0.54

**Table 4:** Means, standard deviations, and Pearson correlations among the observed variables in the Base and Detailed Models.

Variables	Mean	SD	1	2	3	4	5	6	7
1. Positive problem-solving self-efficacy	4.15	0.48	1						
2. Negative problem-solving self-efficacy (reverse-scored)	3.96	0.67	0.38 **	1					
3. Critical thinking disposition	3.90	0.44	0.64 **	0.25 **	1				
4. Mathematical creativity self-efficacy	3.67	0.54	0.54 **	0.34 **	0.63 **	1			
5. Mathematical creativity self-efficacy (Fluency)	3.73	0.57	0.51 **	0.38 **	0.60 **	0.90 **	1		
6. Mathematical creativity self-efficacy (Flexibility)	3.94	0.55	0.56 **	0.33 **	0.60 **	0.88 **	0.75 **	1	
7. Mathematical creativity self-efficacy (Originality)	3.35	0.69	0.38 **	0.22 **	0.49 **	0.89 **	0.67 **	0.64 **	1

*Note. N* = 554. ** *p* < 0.01 (two-tailed). Negative Problem-Solving Self-Efficacy was reverse-scored; therefore, higher scores indicate higher levels of problem-solving self-efficacy.

**Table 5:** Pathways, standardized direct, indirect, and total effect coefficients, and mediation results for the Base Model.

Pathways	Bootstrap Estimates (5000 Resamples)									Conclusion
	Direct Effect (β)	95% CI [LB, UB]	*p*	Indirect Effect (β)	95% CI [LB, UB]	*p*	Total Effect (β)	95% CI [LB, UB]	*p*	
PSEPS → CTD	0.64	[0.56, 0.70]	0.001	-	- -	-	0.64	[0.56, 0.70]	0.001	-
CTD → MCSE	0.48	[0.39, 0.56]	<0.001	-	- -	-	0.48	[0.39, 0.56]	<0.001	-
NSEPS-R → MCSE	0.15	[0.09, 0.23]	<0.001	-	- -	-	0.15	[0.09, 0.23]	<0.001	-
PSEPS → MCSE *(via CTD)*	0.17	[0.10, 0.26]	<0.001	0.31	[0.25, 0.37]	<0.001	0.48	[0.40, 0.55]	<0.001	Partial Mediation

*Note.* PSEPS: Positive problem-solving self-efficacy; NSEPS-R: Negative problem-solving self-efficacy (reverse-scored); CTD: Critical thinking disposition; MCSE: Mathematical creativity self-efficacy; CI: Confidence Interval; LB: Lower Bound; UB: Upper Bound.

**Table 6:** Explained variance (R^2^), confidence intervals (CI), and effect sizes for endogenous variables in the Base Model.

Endogenous Variables	Bootstrap Estimates (5000 Resamples)			Effect Size
	Explained Variance (R^2^)	95% CI [LB, UB]	*p*	
Critical thinking disposition	0.41	[0.31, 0.49]	0.001	Large
Mathematical creativity self-efficacy	0.45	[0.38, 0.51]	0.001	Large

*Note.* LB: Lower Bound; UB: Upper Bound.

**Table 7:** Pathways, standardized direct, indirect, and total effect coefficients, and mediation results for the Detailed Model.

Pathways	Bootstrap Estimates (5000 Resamples)									Conclusion
	Direct Effect (β)	95% CI [LB, UB]	*p*	Indirect Effect (β)	95% CI [LB, UB]	*p*	Total Effect (β)	95% CI [LB, UB]	*p*	
PSEPS → CTD	0.64	[0.56, 0.70]	0.001	-	- -	-	0.64	[0.56, 0.70]	0.001	-
NSEPS-R → MCSE_Ori	0.11	[0.03, 0.18]	0.007	-	- -	-	0.11	[0.03, 0.18]	0.007	-
NSEPS-R → MCSE_Flex	0.14	[0.06, 0.21]	<0.001	-	- -	-	0.14	[0.06, 0.21]	<0.001	-
NSEPS-R → MCSE_Flu	0.22	[0.14, 0.29]	<0.001	-	- -	-	0.22	[0.14, 0.29]	<0.001	-
CTD → MCSE_Ori	0.46	[0.39, 0.53]	<0.001	-	- -	-	0.46	[0.39, 0.53]	<0.001	-
CTD → MCSE_Flex	0.44	[0.35, 0.51]	<0.001	-	- -	-	0.44	[0.35, 0.51]	<0.001	-
CTD → MCSE_Flu	0.48	[0.40, 0.55]	0.001	-	- -	-	0.48	[0.40, 0.55]	0.001	-
PSEPS → MCSE_Ori *(via CTD)*	-	- -	-	0.29	[0.24, 0.35]	<0.001	0.29	[0.24, 0.35]	<0.001	Full Mediation
PSEPS → MCSE_Flex *(via CTD)*	0.21	[0.14, 0.30]	<0.001	0.28	[0.22, 0.34]	<0.001	0.49	[0.42, 0.56]	<0.001	Partial Mediation
PSEPS → MCSE_Flu *(via CTD)*	0.11	[0.03, 0.17]	0.006	0.31	[0.25, 0.37]	<0.001	0.42	[0.34, 0.48]	<0.001	Partial Mediation

*Note.* PSEPS: Positive problem-solving self-efficacy; NSEPS-R: Negative problem-solving self-efficacy (reverse-scored); CTD: Critical thinking disposition; MCSE: Mathematical creativity self-efficacy; MCSE_Ori: Mathematical creativity self-efficacy (Originality); MCSE_Flex: Mathematical creativity self-efficacy (Flexibility); MCSE_Flu: Mathematical creativity self-efficacy (Fluency); CI: Confidence Interval; LB: Lower Bound; UB: Upper Bound.

**Table 8:** Explained variance (R^2^), confidence intervals (CI), and effect sizes for endogenous variables in the Detailed Model.

Endogenous Variables	Bootstrap Estimates (5000 Resamples)			Effect Size
	Explained Variance (R^2^)	95% CI [LB, UB]	*p*	
Critical thinking disposition	0.41	[0.31, 0.49]	0.001	Large
Mathematical creativity self-efficacy (Originality)	0.25	[0.18, 0.31]	0.001	Medium
Mathematical creativity self-efficacy (Flexibility)	0.43	[0.35, 0.49]	0.001	Large
Mathematical creativity self-efficacy (Fluency)	0.42	[0.35, 0.48]	0.001	Large

*Note.* LB: Lower Bound; UB: Upper Bound.

**Table 9:** Parameter estimates for the initially hypothesized direct paths constrained in the final models.

Structural Model	Initially Hypothesized Direct Path	β	S.E.	C.R.	*p*	Decision
Base Model	NSEPS-R → CTD	0.007	0.023	0.302	0.762	Constrained to zero
Detailed Model	NSEPS-R → CTD	0.007	0.023	0.302	0.762	Constrained to zero
Detailed Model	PSEPS → Originality	0.125	0.072	1.737	0.082	Constrained to zero

*Note.* This table reports the parameter estimates for the direct paths initially specified in the hypothesized structural models but subsequently constrained to zero in the final parsimonious models.

**Table 10:** Nested chi-square difference tests comparing the hypothesized and final parsimonious models.

Comparison	χ^2^	df	Δχ^2^	Δdf	*p*	Decision
Hypothesized Base vs. Parsimonious Base	0.091	1	0.091	1	0.762	Parsimonious model retained
Hypothesized Detailed vs. Parsimonious Detailed	3.099	2	3.099	2	0.212	Parsimonious model retained

*Note.* Non-significant Δχ^2^ values indicate that imposing the specified constraints did not produce a statistically significant deterioration in overall model fit.

**Table 11:** Fit comparison of the proposed and reverse-direction competing models.

Model	χ^2^	df	χ^2^/df	CFI	RMSEA (90% CI)	AIC	Support for Theoretical Structural Paths
Base Model (Proposed)	0.091	1	0.091	1	0.000 [0.000, 0.076]	18.091	All paths significant
Base Reverse Model	1.373	1	1.373	0.999	0.026 [0.000, 0.120]	19.373	All paths significant
Detailed Model (Proposed)	3.099	2	1.55	0.999	0.032 [0.000, 0.096]	41.099	All nine paths significant
Detailed Reverse Model	2.599	2	1.3	1	0.023 [0.000, 0.085]	40.599	Four paths non-significant

*Note.* For the Base Models, ΔAIC = 1.28. For the Detailed Models, ΔAIC = 0.50. According to Burnham and Anderson (2002), ΔAIC values of 2 or less indicate substantial empirical support for competing models. Thus, the information criteria provide little basis for distinguishing between the proposed and reverse-direction specifications. This pattern is also consistent with the problem of statistically equivalent or near-equivalent structural models described by MacCallum et al. (1993).

## Data Availability

The raw data supporting the conclusions of this article will be made available by the authors on request.
